# Novel Probiotic/Bacterial Cellulose Biocatalyst for the Development of Functional Dairy Beverage

**DOI:** 10.3390/foods11172586

**Published:** 2022-08-26

**Authors:** Iliada K. Lappa, Vasiliki Kachrimanidou, Maria Alexandri, Aikaterini Papadaki, Nikolaos Kopsahelis

**Affiliations:** Department of Food Science and Technology, Ionian University, 28100 Argostoli, Kefalonia, Greece

**Keywords:** cheese whey, freeze-dried starters, probiotic, cell immobilization, bacterial cellulose, fermented food, functional food

## Abstract

The development of innovative functional products with potential health benefits, under the concept of bio-economy, is flourishing. This study undertook an evaluation of non-dairy lactobacilli *Lactiplantibacillus pentosus* B329 and *Lactiplantibacillus plantarum* 820 as “ready to use” starter cultures. Lactic acid bacteria (LAB) cultures were evaluated for their fermentation efficiency, before and after freeze-drying, using cheese whey (CW) as a fermentation substrate and subsequent immobilization on bacteria cellulose (BC) to produce a novel biocatalyst. The biocatalyst was applied in functional sour milk production and compared with free cells via the assessment of physicochemical and microbiological properties and sensory evaluation. Evidently, LAB strains exhibited high fermentative activity before and after freeze-drying. Results of a 5-month storage stability test showed that viability was 19% enhanced by immobilization on BC, supporting the concept of “ready to use” cultures for the production of fermented beverages. Likewise, sour milk produced by the BC biocatalyst presented higher organoleptic scores, compared to the free cells case, whereas immobilization on BC enhanced probiotic viability during post-fermentation storage (4 °C, 28 days). The obtained high viability (>10^7^ log cfu/g) demonstrated the efficacy of the proposed bioprocess for the production of functional/probiotic-rich beverages. Ultimately, this work presents a consolidated scheme that includes the advantages and the cooperative effect of probiotic LAB strains combined with a functional biopolymer (BC) towards the formulation of novel functional products that coincide with the pillars of food systems sustainability.

## 1. Introduction

The formulation of novel functional ingredients or products with enhanced health benefits constitutes a flourishing and constantly expanding research area [1,2]. Among others, living microorganisms exhibit a critical role in these products, opting to meet the demands of consumers and the food industry. Likewise, LAB have widely demonstrated the ability to confer significant characteristics on fermented milk products, including unique organoleptic characteristics and potential health benefits [3,4,5,6,7]. In the context of technological applications of LAB cultures, delivery strategies able to extend microbial viability, prolong shelf life, and increase product stability during storage are at the forefront of attention [8]. Probiotics are defined as *“live microorganisms which when administered in adequate amounts confer a health benefit on the host”* [9]. Enhanced probiotic viability continues to be the primary target in the development of functional dairy products. Currently, numerous different approaches have been employed to sustain and promote microbial cell viability through strain selection, immobilization techniques, or symbiotic inclusion [10,11,12,13]. Regarding microbial immobilization, various matrices, including composite carrier systems, have received significant attention as suitable support materials towards cell protection under diverse stress conditions such as heat treatment or acidic environment [14,15,16].

BC is a functional microbial polymer and is a type of dietary fiber as it consists of β (1→4)-linked glucose units that self-assemble into fibers. However, its production process (from molecular regulation of intracellular synthesis, extracellular transport, and organization of cellulose into highly ordered structures) is a quite complicated phenomenon [17]. The USA Food and Drug Administration has classified BC as GRAS (Generally Recognized As Safe) based on its nontoxic, biocompatible, biodegradable, and bioabsorbable nature [18,19]. BC production has already been demonstrated via bioconversion processes by microbial and cell-free enzymes systems, using textile wastes and several agro-industrial wastes and by-products (e.g., fruit waste, brewery waste, raisin by-products, and whey), thus contributing to process sustainability [20,21,22,23,24].

Currently, the growing consumer awareness toward diet modification and the environment indicates a strong demand for sustainable food and beverage production. Likewise, technologies and studies dealing with the exploitation of renewable resources and environmentally benign processing, fostering food preservation and nutritional value, are lately gaining significant attention.

In this sense, over the last decade, scientific and industrial research has focused on the valorization and commercialization of an abundant and low-cost renewable resource of the dairy industry; namely, cheese whey. To decontaminate its pollution stream, several effective biotransformation processes to generate a variety of added-value products have been developed and proposed [25]. The primary purpose is to use cheese whey lactose, as a carbon source, to replace the conventional and expensive synthetic media that are usually applied in bioconversion processes. On top of that, the expanding interest for whey proteins enables circular economy strategies [25,26]. According to recent market reports, worldwide permeate market (obtained from milk or whey) is expected to reach US $1.29 billion by 2032, driven by the increasing demand for it as a food supplement [27]. The cost of unprocessed cheese whey permeate (CWP) is expected to be between 1 and 1.5 cents per liter. Simultaneously, the global probiotics market is reported to also escalate to more than US $110 billion by 2030, demonstrating a CAGR (compound annual growth rate) of 7.5% between 2021–2030 [28]. Studies dealing with whey utilization for the production of microbial/probiotic biomass are constantly rising, as the high lactose content of whey makes it a suitable substrate for microbe culture [29,30,31]. In terms of lactose-to-biomass conversion efficiency, the formation of starter cultures on a low-cost non-synthetic substrate is critical for commercial-scale culture production and their subsequent application in the food sector. The aforementioned approach designates a realistic and cost-effective technique that could be conveniently adopted towards the manufacture of functional and sustainable food products.

The current study introduces the development of “ready to use” freeze-dried LAB cultures using cheese whey as fermentation feedstock and the subsequent formulation of a novel BC-based biocatalyst. The obtained probiotic/cellulose biocatalyst was further employed in the production of functional fermented milk, followed by evaluation of product microbial and sensory characteristics. Ultimately, the present study describes the development of a novel biocatalyst that assures sufficient probiotic populations and advantageous organoleptic characteristics, and hence contributes towards the establishment of functional formulations and sustainable food systems.

## 2. Materials and Methods

### 2.1. Bacterial Strains and Culture Conditions

Two different LAB strains were screened for their ability to ferment lactose prior to the evaluation of further applications. Strains originally belonging to the culture collection of the Agricultural University of Athens (Laboratory of Food Microbiology and Technology and Laboratory of Food Quality Control and Hygiene) were kindly provided for this study. Specifically, *L. pentosus* FMCC B329, previously isolated from olive brine, was used as probiotic type strain [32]. In addition, *L. plantarum* LQC 820 isolated from sausages was used as a starter and potential probiotic strain. LAB cultures were stored at −80 °C in 20% glycerol (*v*/*v*) and were routinely activated in de Man, Rogosa, and Shape broth (MRS) (Difco, Belgium) for 24 and 16 h subsequently (time required for exponential growth phase) at 37 °C prior to use.

### 2.2. Submerged Fermentations

CW was provided by a local dairy plant (Galiatsatos, Kefalonia, Greece). Prior to designated use, whey was deproteinized to avoid protein precipitation at acidic fermentation conditions, which interferes with biomass measurements [33]. Briefly, the pH value was adjusted to 4.5 (using 5 N HCl), and the mixture was heated (100 °C, 10 min) for protein denaturation. The precipitated protein was removed by centrifugation (7.000 rpm, 15 min), the pH was adjusted to 6.5, and the lactose-rich derived stream was autoclaved (121 °C/ 15 min) before further use. The obtained cheese whey permeate (CWP) (~50 g/L lactose) was supplemented with yeast extract (5 g/L), K_2_HPO_4_ (1 g/L), (NH4)_2_SO_4_ (5 g/L) and MgSO_4_•7H_2_O (0.3 g/L).

Submerged fermentations were conducted in 1 L Duran bottles containing 500 mL of whey media and 5% (*v*/*v*) of LAB pre-cultures were used to inoculate the substrate. Experiments were carried out at 37 °C under static conditions and pH was manually adjusted (using 5 N KOH) throughout the fermentation process. Fermentation kinetics for lactose assimilation and lactic acid production were recorded by high-performance liquid chromatography (HPLC) analysis (1200 series Agilent Technologies, Santa Clara, CA, USA) as previously described [21]. Dry cell weight (DCW) was determined at 6 h intervals. Samples were centrifuged at 7000 rpm for 10 min, the supernatant was discarded, and precipitated cells were washed twice with deionized water to remove substrate residues. The collected biomass was dried at 100 °C until the weight was stabilized. After drying, samples were weighed, and the results were expressed in g/L. All experiments were carried out in duplicate.

### 2.3. Freeze-Dried Biomass Production

Following Section 2.2, cell mass was harvested by centrifugation at 7000 rpm for 10 min at 5 °C, and cells were collected after double-rinsing with Ringer’s solution (¼ strength) to remove substrate residues and fermentation by-products. Solutions of sucrose (2% *w*/*v*) and whey protein (5% *w*/*v*) were added to the cells as cryoprotectants [34,35]. Lyophilization process was carried out under vacuum in a freeze-drying system (BK-FD12P, Biobase Biodustry Co Ltd., Jinan, China) under the following conditions: cooling temperature −60 °C, pressure of 0.08 mbar, and lyophilization time of 24 h. The freeze-dried strains were placed in sealed vials and kept at 4 °C in a light-protected environment.

Subsequently, the freeze-dried cells were compared to wet cultures with respect to their viability and fermentation efficiency. Duplicates of each strain were subjected to serial dilutions and the viable counts (cfu/g) were enumerated after plating on double-layer MRS agar and incubating at 37 °C for 48 h.

### 2.4. Immobilized Biocatalysts Production

BC was produced as described in our previous study [21] by *A. xylinum* using hydrolyzed cheese whey as a fermentation substrate. The produced cellulose was initially used in its original form as a film membrane, while in the latter experiments, to produce sour milk, films were cut into small pieces (~5 mm) using a domestic blender, and cellulose was collected after centrifugation (7000 rpm, 10 min). Both forms of cellulose were sterilized for 15 min at 121 °C prior to use. Immobilization in BC was performed by the ‘adsorption-incubation’ method slightly modified [36]. Briefly, 10 g (on wet base) of BC was added to deproteinized whey and the system was left for 12 h at 37 °C. Wet and freeze-dried cultures of the strains *L. pentosus* and *L. plantarum* were used to evaluate their immobilization ability. Liquid cultures kept at −80 °C were refreshed on MRS substrate at 37 °C before use, whereas freeze-dried cultures kept at 4 °C were added directly to deproteinized whey. The mixture was stirred at regular times intervals in order to distribute the carrier and the microorganisms evenly. After immobilization, the respective biocatalysts were separated from the substrate under aseptic conditions and immersed twice in sterile Ringer’s solution to remove media and slightly adherent cells. The corresponding microbiological analyses were performed to evaluate the successful immobilization of the cells. More specifically, for the enumeration of LAB, 10 g of each immobilized biocatalyst was homogenized with 90 mL of Ringer’s solution followed by serial dilutions. Suspensions were incorporated into MRS plates for viable cell count determination. The number of populations was expressed as log cfu/g BC.

The produced biocatalysts were reactivated in fresh deproteinized whey after storage at 4 °C for 5 months. Cell survival was calculated as: % viability = (N/No) × 100, where N and No are the number of cells per gram of biocatalyst after and before storage, respectively [37]. Storage stability of lyophilized cultures was also monitored and expressed as log cfu/g of dried powder.

### 2.5. Sour Milk Production

Commercial homogenized–pasteurized cow milk (sugars 4.6%, fat 3.5%, protein 3.2%, pH 6.8) was used for the production of fermented milk. The produced biocatalyst was immersed in sterile glass containers containing 200 mL milk and left at 37 °C until the pH reached 4.5. Likewise, free LAB cells (10^8^–10^9^ cfu/mL) were also inoculated in milk, to perform the same fermentation process. After the pH value dropped to 4.5, sour milk was quickly cooled to 4 °C in an ice water bath. All samples were stored at 4 °C up to 4 weeks for further analysis.

### 2.6. Microbiological Analysis

The viability of *L. pentosus* and *L. plantarum* was evaluated at specific time intervals (1, 7, 14, 21, and 28 days) by collecting 10 g of samples. The enumeration was performed as described above (Section 2.2). Potential spoilage microbiota growth was also assessed. In particular, yeast and molds were determined by plating on PDA (potato dextrose agar) after incubation at 30 °C for 72 h and coliforms were detected on VRBA (violet red bile agar) after incubation at 37 °C for 24 h. All the above media were sourced from Condalab (Madrid, Spain).

### 2.7. Physicochemical Analysis

The pH value of the fermented milk was measured by a digital pH meter (HI 98100, Hanna, Woonsocket, RI, USA) at specific time intervals (1, 7, 14, 21, 28 days). Titratable acidity was assessed using 0.1 mol/L NaOH and phenolphthalein as an indicator and expressed as lactic acid per 100 g of sour milk.

Susceptibility of sour milk to syneresis was measured using a previously described method [38]. Particularly, 2 g of each sample was weighed and centrifuged at 5000 rpm for 10 min at 4 °C. Syneresis % was determined as (Ws × 100)/Wg (Ws = weight of supernatant after centrifugation; Wg = the total weight of the sample).

### 2.8. Sensory Evaluation

Sensory analysis of the produced sour milk was performed on the 14th day of storage by 10 panelist-laboratory members (5 males and 5 females, aged 25–45 years old, with chemical, biological, or food technology scientific backgrounds), familiar with fermented milk consumption. The organoleptic assessment was in line with previous studies [11]. Randomized samples (50 mL) were evaluated for color, sour odor, flavor, smoothness, and overall acceptance using a common nine-point hedonic scale ranging from 1–9 (1 = extremely disliking and 9 = extremely liking). Commercial products were also used for comparison reasons.

### 2.9. Statistical Analysis

The results were analyzed by ANOVA and statistical significance was established at *p* < 0.05. In particular, analysis of variance (one-way ANOVA) was applied to estimate any significant differences among BC immobilization treatments and strains viability. Similarly, the microbiological and physicochemical results of fermented milks during storage were assessed for significant variances using post-hoc Tuckey HSD.

## 3. Results

### 3.1. Evaluation of Probiotics on CWP Fermentations

**Figure 1:**
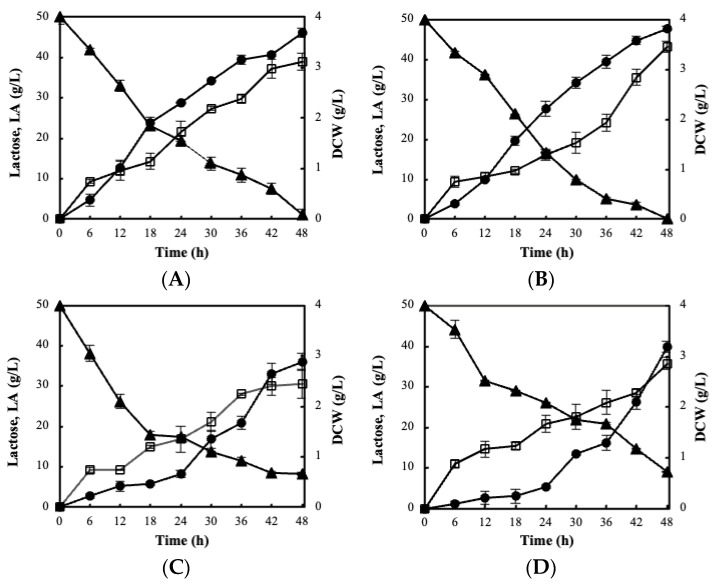
Profile change of (□) dry cell weight (DCW), (●) lactic acid (LA), and (▲) lactose of *L. pentosus* B329 (**A**) and *L. plantarum* 820 (**B**) wet cultures, *L. pentosus* B329 (**C**) and *L. plantarum* 820 (**D**) freeze-dried cultures during CWP fermentation of ~50 g/L initial lactose concentration at 37 °C.

The first step of the experiments employed the fermentation evaluation of the two non-dairy strains, *L. pentosus* B329 and *L. plantarum* 820, using CWP as a low-cost substrate. In fact, both strains were able to assimilate up to 98% of the initial lactose, whereas supplementation of CWP demonstrated significant potential for cell mass production ranging between 3.12 and 3.46 g/L (Figure 1A,B).

Lactose consumption occurred in parallel with biomass production, whereas lactic acid production reached approximately ~45 g/L. Notably, 61–66% of lactose was consumed during the first 24 h of fermentation, resulting to complete assimilation at 48 h. The pH value decreased significantly at the beginning of the fermentations, indicated by a rapid shift (pH < 4.5) during the first 6 h of incubation.

Based on these results, both strains (*L. pentosus* and *L. plantarum*) were further employed to evaluate their fermentation capacity and functional stability after freeze-drying. Worth noting, the protein stream of cheese whey was applied as cryoprotectant, aiming to utilize both streams of CW. The population levels of both strains remained stable before and after lyophilization (~10^10^ cfu/g). Further on, the metabolic stability of freeze-dried cultures was compared to wet cultures in a similar whey fermentation process. The results revealed that lactose assimilation was quite decreased, since freeze-dried cultures of *L. pentosus* B329 and *L. plantarum* 820 fermented around 83% and 82% of the original substrate, respectively (Figure 1C,D). Likewise, a decrease in lactic acid production was also detected for both strains. Finally, a reduction in the final produced biomass of approximately 20% was noticed in both strains compared to the wet cultures.

### 3.2. Fermentation Kinetics, Bacterial Viability and Storage Stability Using the BC/Biocatalyst

**Figure 2:**
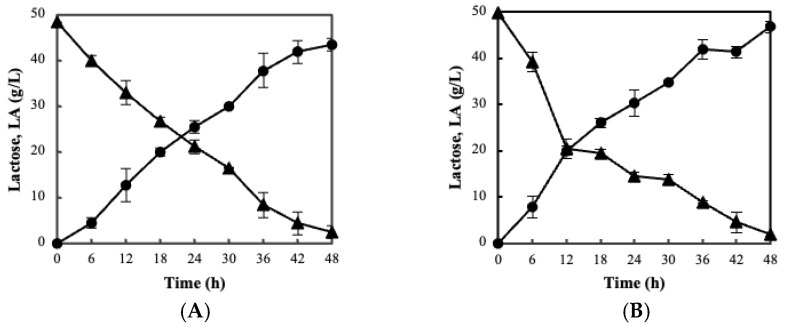
Kinetics of lactose (▲) and (●) lactic acid (LA) during CWP fermentation by BC-immobilized strain *L. pentosus* B329 (**A**) and *L. plantarum* 820 (**Β**) at 37 °C.

**Figure 3:**
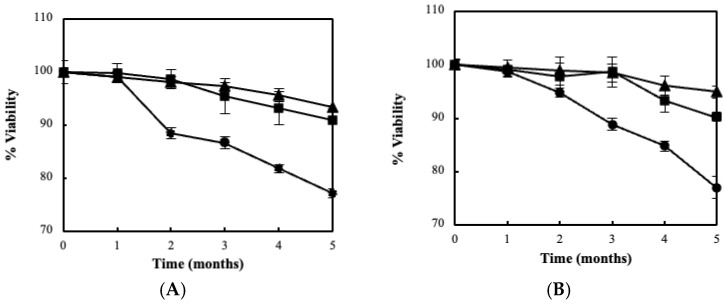
Viability of free (●) and immobilized cells of *L. pentosus* B329 (**A**) and *L. plantarum* 820 (**B**) on bacterial cellulose (▲: BC film and ■: blended BC) during 5 months of storage at 4 °C.

Both aforementioned cultures were subsequently immobilized using the developed BC matrices. Both forms of BC employed were proved to be suitable carriers for LAB immobilization as validated by the enumerated microbial cells. According to Table 1, the freeze-drying process did not significantly affect the immobilization efficacy of bacteria (*p* > 0.05). In particular, a large population of *L. pentosus* B329 cells is shown to be attached on film and blended BC ranging between 8.74 and 8.06 log cfu/g, respectively. Similarly, the population of *L. plantarum* 820 was 8.58 and 8.43 log cfu/g for film and blended BC, respectively. Ιt is worth noting that significant differences occurred in the final cell attachment between the two types of BC that were used (*p* < 0.05). The evaluation of the fermentation ability of the novel biocatalyst was also performed on CWP, and lactose assimilation was assessed. As a matter of fact, the fermentation kinetics of the produced biocatalysts revealed enhanced lactose assimilation ranging between 95.2% for *L. pentosus* B329 and 96.3% for *L. plantarum* 820 (Figure 2). Furthermore, the biocatalysts proved to be stable during long-term storage at 4 °C in whey, maintaining high numbers of viable cells over the required limit for probiotic products (>7 log cfu/g). As clearly demonstrated in Figure 3, cell immobilization on BC had a significantly positive effect on the viability of both strains (*p* < 0.05), while no intra-species differences were observed. The viability levels after a 5-month storage period ranged from 90 to 95%, whereas the respective levels of free cells were lower than 80%.

### 3.3. Sour Milk Production Using Probiotic Cellulose Biocatalyst

**Figure 4:**
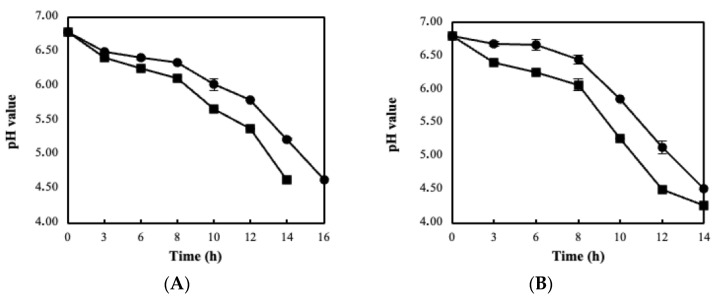
Milk acidification profile using free (●) and immobilized biocatalysts on blended BC (■) of the strains *L. pentosus* B329 (**A**) and *L. plantarum* 820 (**B**).

**Figure 5:**
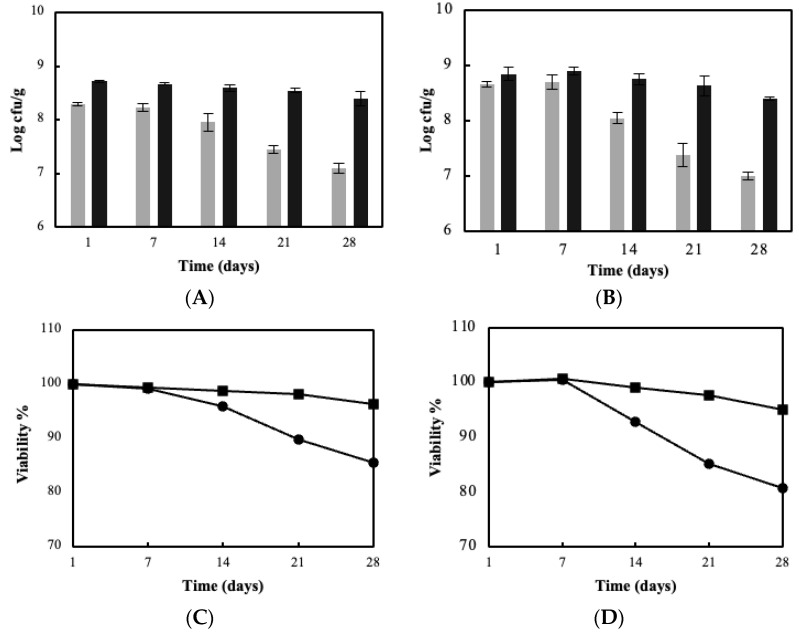
Populations (■: free cells, and ■: blended BC) and viability of *L. pentosus* B329 (**A**,**C**) and *L. plantarum* 820 (**B**,**D**) in sour milk produced using free cells (●) and blended BC immobilized biocatalyst (■) during post-fermentation storage for 28 days (4 °C).

The developed “ready to use” biocatalyst were further on assessed for fermented milk production (sour milk). For technological reasons, only the blended BC biocatalyst was applied and compared with the free cells case. Figure 4 displays the ability of *L. pentosus* and *L. plantarum* biocatalysts to be used as starter cultures. Milk fermentations, either by free or immobilized LAB strains, led to a pH drop, reaching final values of 4.64 and 4.26 respectively and hence to the formulation of sour milk. It can be easily observed that immobilization in BC enhanced the acidification rate for the two *Lactobacillus* strains compared to free cells, without significantly affecting the total fermentation time. Further on, the successful incorporation of probiotic or adjunct cultures into sour milk is primarily determined by the cells’ ability to survive in the resulting acidic conditions during storage, thus populations were monitored at 7-day intervals. As reported in Figure 5A, the blended BC biocatalyst retained high viable counts of >8 log cfu/g during the storage period. When free cells were applied, viable numbers were significantly lower (~7.0 log cfu/g) with respect to the immobilized biocatalyst in both strains (*p* < 0.05). Likewise, the % viability of immobilized cells follows a similar trend (Figure 5B). In particular, decreased viability was observed in the case of free cells (80%), whereas the respective percentage for blended BC was 95%, on the 28th day of storage.

The physicochemical characteristics of the fermented milks using free cells and immobilized biocatalyst after 28 days of storage are summarized in Table 2. Titratable acidity and pH values were significantly affected (*p* < 0.05) by storage time, since post-acidification was observed in all produced samples. Particularly, pH values ranged in the levels of 3.53–4.52 in the case of blended BC. Similar observations are presented for titratable acidity profile during storage, whereas significant differences between the two LAB strains were also evidenced (*p* < 0.05).

**Figure 6:**
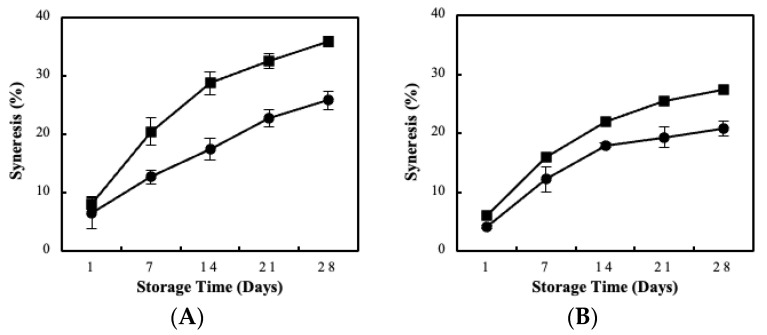
Syneresis changes of sour milk produced by free (●) and blended BC immobilized biocatalyst (■) of *L. pentosus* B329 (**A**) and *L. plantarum* 820 (**Β**) during storage in 28 days (4 °C).

The percentage (%) syneresis of sour milk samples is illustrated in Figure 6. Storage time significantly affected syneresis in all sour milk samples whereas the increase is speculated to relate with the pH drop and the acidity rise (*p* < 0.05) [39]. Both strains exhibited higher syneresis values by the use of the blended BC in comparison with the case of free cells. This could be explained by the increased metabolic activity provided by the biocatalyst, which lowered the pH values, as also observed during post-acidification kinetics (Table 2). Moreover, variances were observed between the two LAB strains ranging from 25–36% for the biocatalyst cases and 20–25% for the free cell cultures cases. Syneresis for all samples was also observed macroscopically on the last day of storage. However, sour milk samples were reformed into a homogenous structure after shaking, thus resembling the commercial sample.

Finally, sensory evaluation was conducted for all the produced fermented milks, in comparison with a commercial product (Table 3). In general, the first group of sour milk using *L. pentosus* B329 received lower score values compared to the second group of milks using *L. plantarum* 820. Notably, the fermented milk produced by the immobilized biocatalyst of blended BC received higher score values compared to that produced by free cell cultures. Samples produced by both strains were characterized by a pleasant texture. However, a sweeter odor was observed, explaining the lower score values of sour odor. In terms of overall acceptance, no significant differences were observed between the second group and the commercial sour milk. The addition of immobilized *L. pentosus* B329 and *L. plantarum* 820 cells received similar ratings regarding samples containing BC (B2).

## 4. Discussion

The development of sustainable bio-based processes to generate added-value products is constantly expanding. Food industry waste and by-product streams have been previously used for LAB biomass production [40]. Likewise, *L. plantarum* and *L. pentosus* constitute two of the most extensively employed species [30,41,42]. Several reports have evidenced the successful implementation of CW for the proliferation and growth of LAB [43,44]. Several researchers also report the necessity to implement synthetic exogenous sources, to boost microbial growth, achieving cell growth equal or higher than that of synthetic substrates [8,45]. For instance, Prajapati et al. [42] presented whey fermentation by *L. helveticus* using additional nitrogen sources, e.g., peptone and yeast extract, leading to 3.25 g/L biomass production. Similar findings have also been reported, with *L. casei* in whey, demonstrating significant lactose conversion (95.62%) and lactic acid production (33.73 g/L) [46]. The use of LAB cultures in industrial food processing encompasses not only microbiological but also technological and economical aspects. Nowadays, freeze-drying remains one of the most applicable and effective approaches for probiotic stabilization and functional food production [47,48,49]. In addition, the high viability levels found in this study are expected to be replicated, as the use of whey protein (WP) stream as a cryoprotectant has being already established as a beneficial practice [50,51]. In fact, utilizing WP confers multiple advantages for industrial applications since it eliminates the risk of adverse quality effects on the produced beverages and endorses the nutritional value of the final product. Thus, our study proposed an additional route to valorize cheese whey streams to generate value-added products as potential food supplements but also to support dairy waste management.

Studies on the development of an efficient probiotic or adjunct biocatalyst involve the assessment of the interdependence between cellular stability and metabolic activity. On the other hand, cell immobilization has been widely recognized as a way to modulate catalytic processes and enhance the fermentation performance in several areas, including bioprocessing, food, and pharmaceuticals, among others. Likewise, BC has been evaluated for enzyme immobilization with relatively low efficiency, hence necessitating activation techniques to modify its sophisticated three-dimensional structure [52,53]. In addition, the suitability of BC to be used as a potential cell immobilization matrix with unique properties was recently reported [19,54]. However, scientific data on the behavior and physiology of bacteria in the immobilized state are still limited compared to natural environments, where cells usually adhere to surfaces, entailing biofilm formation. In this frame, BC has not yet been established as a probiotic biocatalyst for functional food production. This study indicated the development of a delivery vehicle able to ensure sufficient probiotic populations until the time of consumption, as proven by the clear persistence of high survival rates during both drying and storage. It is noteworthy that in the case of BC biocatalyst application, high viability levels were observed for both strains. Similarly, Phrompeth et al. [55] reported that *L. plantarum* cells immobilized on BC retained their viability, at a high level, during storage in the acidic environment of mamao juice, while Fijałkowski et al. [56] concluded that immobilization of LABs on BC provides high-level protection of the microorganisms against the influence of gastric juices and bile salts. Immobilization studies conducted in the present work, displayed a high efficacy of immobilization evidenced by cell viability (>8 log cfu/g). More specifically, the ‘absorption-incubation’ method, used in this study, has been already proved to promote cell proliferation and invasion across the entire cellulose matrix according to Savitskaya et al. [57]. In addition, BC is a complex fibril network forming pores with dominant size of 5–8 μm, as obtained in our previous study [58]; being in line with Gao et al. (2011) who reported that BC forms a porous network with an average size of 10 μm and surface area of about 86 m^2^/g [59]. Considering that lactobacilli are rodlike bacteria with a length of around 1–1.5 μm and a diameter of 0.7–1 μm [60], BC seem to be a suitable natural immobilization matrix.

Immobilized systems are often reported to outperform free cell cultures in a number of ways, including increased resistance to acidic conditions and increased biocatalytic efficiency [61]. Ιn line with our findings, Dimitrellou et al. [39] also reported higher acidification activities when *L. casei* cells immobilized on apple pieces were compared to freeze-dried free cells. In general, the development of functional foods by LAB using a wide range of immobilization supports is flourishing. Ultimately, however, viability is generally considered a prerequisite of paramount importance, hence defining the true industrial challenge. According to the US FDA (United States Food and Drug Administration), a minimum of 6 log cfu/g of probiotic and adjunct cultures in food is required [62]. Similarly, Terpou et al. [38] detected high storage stability of *L. casei* immobilized in wheat bran, regardless the removal of the biocatalyst at the end of fermentation.

In terms of product development, our results indicated that free and immobilized *Lb. pentosus* and *L. plantarum* strains had no adverse effect on the physicochemical properties of fermented milk or negative impact on the sensory profile of the formulated product. In particular, physicochemical characteristics were affected by storage time in correlation with a previous study using freeze-dried *L. casei* cells [39]. Sensory evaluation revealed that the produced fermented dairy beverages scored similarly to their commercial counterparts after 21 days in cold storage, even though they were found to be less acidic and had a sweeter taste than the commercial versions, as reported in a similar study [38]. BC was employed as an edible food ingredient without impairing the sensory properties of the resulting sour milk, such as flavor or color. Generally, most substrates that are utilized as probiotic carriers are not resistant to digestion. Nevertheless, BC has been already reported to be used as a source of dietary fiber for the manufacturing of several products, such as desserts or fruit cocktails, providing health benefits [63]. Overall, it is evident that the application of BC in fermented sour milk production had a positive effect on product stability and consumer acceptance, whereas BC proved to be an excellent carrier of probiotic bacteria.

## 5. Conclusions

The present work demonstrated that LAB strains *L. pentosus* B329 and *L. plantarum* 820 can be successfully used as starter cultures for sour milk production, presenting high survival rate during storage and providing potential probiotic effects. Immobilization of LAB strains on BC sustained cell viability and hence their metabolic activity as evidenced by cell proliferation and lactic acid production. The obtained results are of paramount importance particularly when potential probiotic strains are considered to be further implemented as “ready to use” starter cultures. On top of that, sensory characterization of the produced sour milk with blended BC prevailed against the sour milk produced with free cells, and reinforced the concept of formulating novel functional beverages, considering the nutritional value of BC, which is high in dietary fiber. Overall, this study proposed the configuration of a biorefining approach linking the benefits of functional LAB cultures of both dairy and non-dairy origin into an edible natural bio-polymer to formulate innovative fermented products that could confer health benefits through diet modification.

## Figures and Tables

**Table 1:** Immobilized LAB population in different BC forms.

Immobilization Substrate	Strains	log cfu/g (Wet Culture)	log cfu/g (Freeze-Dried Culture)
BC film	B329	8.80 ± 0.09 ^a^	8.74 ± 0.06 ^a^
blended BC	B329	8.33 ± 0.04 ^b^	8.06 ± 0.03 ^b^
BC film	820	8.83 ± 0.05 ^a^	8.58 ± 0.37 ^a^
blended BC	820	8.78 ± 0.28 ^b^	8.43 ± 0.05 ^b^

^a,b^ Similar superscript letters in each column indicates no difference between the mean microbial counts for the two different BC forms.

**Table 2:** The pH and titratable acidity during post-fermentation storage of the sour milk produced by free and immobilized (on blended BC) cells of *L. pentosus* B329 and *L. plantarum* 820 (4 °C).

Storage Time	Free Cells		Blended BC	
	*L. pentosus*	*L. plantarum*	*L. pentosus*	*L. plantarum*
***pH value***				
1	4.50	4.40	4.52	4.22
7	4.46	4.12	4.44	3.82
14	4.44	4.07	4.41	3.75
21	4.35	3.94	4.37	3.56
28	4.31	3.90	4.23	3.53
***Titratable Acidity (% expressed as Lactic Acid)***				
1	0.58	0.60	0.63	0.66
7	0.64	0.67	0.69	0.70
14	0.66	0.71	0.72	0.82
21	0.68	0.82	0.76	1.10
28	0.71	1.02	0.88	1.24

Standard deviation was <5% of the respective values of pH and titratable acidity.

**Table 3:** Sensory evaluation of sour milk produced by LAB strains.

***L. pentosus* B329**	**Color**	**Sour Odor**	**Flavor**	**Smoothness**	**Overall Acceptance**
**C1**	8.2 ± 0.2 ^a^	7.2 ± 0.4 ^a^	6.9 ± 0.2 ^a^	7.6 ± 0.4 ^a^	7.5 ± 0.3 ^a^
**B2**	8.1 ± 0.2 ^a^	7.0 ± 0.3 ^a^	7.6 ± 0.5 ^ac^	8.4 ± 0.2 ^c^	7.6 ± 0.2 ^ab^
***L. plantarum* 820**	**Color**	**Sour Odor**	**Flavor**	**Smoothness**	**Overall Acceptance**
**C1**	8.9 ± 0.2 ^b^	8.4 ± 0.5 ^a^	8.0 ± 0.3 ^a^	8.6 ± 0.4 ^ad^	8.4 ± 0.2 ^a^
**B2**	8.9 ± 0.2 ^b^	7.8 ± 0.5 ^a^	7.5 ± 0.3 ^ab^	8.7 ± 0.3 ^a^	8.2 ± 0.2 ^a^
**Commercial**	8.8 ± 0.2 ^b^	8.5 ± 0.3 ^a^	8.7 ± 0.2 ^d^	8.8 ± 0.2 ^d^	8.6 ± 0.2 ^b^

C1: freeze-dried free culture, B2: immobilized biocatalyst on blended BC. ^a–d^ Means with different superscripts within a column for each LAB strain differ significantly (*p* < 0.05).

## Data Availability

All data are contained within the article.
